# Genome-wide identification and light-quality-dependent characterization of Glutathione S-transferase genes involved in anthocyanin accumulation in *Rhododendron*

**DOI:** 10.1186/s12870-026-08698-1

**Published:** 2026-04-02

**Authors:** Gaoyuan Hu, Yonghong Jia, Yuhang Jiang, Binying Sun, Chenxin Xie, Haichao Hu, Xiaohong Xie, Yueyan Wu

**Affiliations:** College of Biological and Environmental Sciences, Zhejiang Wanli University, Ningbo, Zhejiang 315100 China

**Keywords:** Glutathione S-transferase, GST gene family, *Rhododendron*, light quality, anthocyanin, gene expression

## Abstract

**Background:**

Glutathione S-transferases (GSTs) mediate vacuolar sequestration of anthocyanins and are key determinants of flower color. Several Phi (GSTF) and Tau (GSTU) members have been linked to anthocyanin transport, but how GST repertoires in woody ornamentals respond to light quality is largely unknown in *Rhododendron*.

**Results:**

We identified 87 *GST* genes in the *Rhododendron simsii* genome and classified them into seven subfamilies, with Tau and Phi predominating. Segmental duplication was the main driver of family expansion, and most promoters carried multiple light-responsive cis-elements. Twelve representative *RsGST* homologues were mapped to *Rhododendron hybridum* and showed distinct tissue- and stage-specific expression, including a group (*RsGSTF2*, *RsGSTL7*, *RsGSTU1*) preferentially expressed in floral organs. Under defined light-quality treatments, *GST* transcripts in buds were rapidly induced by UV-A/blue light, whereas red/far-red light elicited stronger and more sustained induction in fully open flowers. Total petal anthocyanin content increased markedly under red and far-red light, broadly paralleling the expression of *RsGSTF2*, *RsGSTL7* and *RsGSTU1*.

**Conclusions:**

Our integrative genomic and expression analyzes indicate that the GST family in *Rhododendron* is tightly connected to light signaling and floral development, and highlight flower-preferential, light-quality-responsive Tau and Phi members as candidate vacuolar anthocyanin transporters for light-dependent flower color formation.

**Supplementary Information:**

The online version contains supplementary material available at 10.1186/s12870-026-08698-1.

## Background

Glutathione S-transferases (GSTs) form a large multifunctional protein superfamily in plants, where they contribute to detoxification, redox homeostasis and the transport of diverse metabolites [1–3]. Plant GSTs are typically divided into several cytosolic subfamilies, among which the plant-specific Phi (GSTF) and Tau (GSTU) classes have undergone marked expansion and are often transcriptionally induced by biotic and abiotic stresses [4–6]-wide surveys in *Arabidopsis*, rice, grape, apple and other crops consistently reveal dozens of GST loci with conserved intron–exon organizations and motif signatures within each subfamily but substantial diversification between subfamilies [7, 8]. Their classical catalytic functions as glutathione transferases or peroxidases, many Phi and Tau GSTs show high affinity for phenolic ligands and have been co-opted as non-enzymatic carrier proteins in specialized metabolic pathways, including flavonoid and anthocyanin metabolism [9–11].The best-characterized examples are the *Zea mays Bronze-2 (Bz2)*, *Petunia hybrida AN9* and *Arabidopsis thaliana TT19* proteins, which are required for vacuolar sequestration of anthocyanins and proanthocyanidins by forming soluble glutathione-independent complexes that are subsequently recognized by membrane transporters [12–14].Orthologous or functionally analogous Phi/Tau GSTs have been functionally characterized as anthocyanin transporters in several fruit crops, where alterations in their expression or coding sequence strongly affect foliage and fruit coloration [15–17].Anthocyanin-related GSTs have also been reported in peach, cultivated strawberry and Chinese bayberry, reinforcing the idea that GST-mediated vacuolar loading represents a conserved module shaping color intensity and patterning in horticultural crops [18–20].Collectively, these studies highlight Phi and Tau GSTs as key modulators of visible pigmentation whose expression patterns and regulatory diversity are likely to contribute to natural variation in flower and fruit color [21–23].

Anthocyanins are major vacuolar pigments responsible for red, purple and blue hues in flowers, fruits and some vegetative organs, and their biosynthetic pathway from phenylalanine via chalcones, flavanones and dihydroflavonols has been extensively elucidated [9, 24, 25].The expression of structural genes encoding enzymes such as CHS, DFR, ANS and UFGT is largely controlled by MYB–bHLH–WD40 (MBW) transcriptional complexes that integrate developmental cues and environmental stimuli [21, 26, 27].Vacuolar sequestration of anthocyanins as glycosylated and often acylated derivatives is critical for their stability, reducing cytosolic toxicity and enabling long-term color display in reproductive organs [11, 23, 28].Current models propose that anthocyanins synthesized at the cytosolic face of the endoplasmic reticulum are transferred to the tonoplast through a coordinated transport module comprising cytosolic GST carrier proteins, ATP-binding cassette (ABC) transporters and multidrug and toxic compound extrusion (MATE) antiporters [11, 12, 14].Mutations in *Bz2*, *AN9*, *TT19* or their orthologues lead to reduced vacuolar pigmentation and the accumulation of unstable anthocyanins in the cytosol, emphasizing the importance of the transport step in linking biosynthetic capacity to visible color phenotypes [13, 15, 18].Genome-wide analyses indicate that plant genomes typically harbor dozens of *GST* genes, but only a small subset of Phi and Tau members appear to have specialized for anthocyanin binding and transport [4, 7, 8].Moreover, promoter analyzes and expression profiling suggest that these anthocyanin-related GSTs are embedded in complex regulatory networks responsive to hormonal and environmental signals, yet their genomic organization and regulatory diversity are still incompletely characterized in woody ornamentals [5, 6, 29].

Light is a major environmental factor controlling anthocyanin accumulation and flower color, acting at multiple levels from the transcription of biosynthetic genes to the regulation of upstream signaling hubs [10, 21, 22] perceive and decode incident light through several classes of photoreceptors, including red/far-red-absorbing phytochromes, blue/UV-A-sensing cryptochromes and phototropins, and the UV-B receptor UVR8, which converge on transcription factors such as HY5 to modulate phenylpropanoid metabolism [30–32].Changes in light intensity and photoperiod can therefore alter anthocyanin biosynthesis by influencing MBW complex activity and hormone signaling, leading to characteristic seasonal or canopy-dependent pigmentation patterns [21, 33, 34].In addition to light quantity, light quality—defined as the spectral composition of incident light—has emerged as a key determinant of anthocyanin profiles, with blue and UV-A wavelengths generally promoting rapid pigment induction, whereas red and far-red wavelengths often exert sustained or stage-specific effects via phytochrome-dependent pathways [35–37].Manipulation of spectral quality using LEDs or filters has demonstrated that red/blue combinations or supplemental blue light can strongly enhance anthocyanin accumulation and modify expression of structural and regulatory genes in lettuce, strawberry, peach, blueberry and tomato fruits [35, 38, 39].Nevertheless, most light-quality studies have focused on biosynthetic enzymes and transcription factors, and much less attention has been paid to downstream transport components such as GSTs, ABC transporters and MATE proteins [10, 11, 40].In particular, the wavelength-specific expression patterns of Phi and Tau GSTs and the extent to which light quality modulates GST-mediated vacuolar loading of anthocyanins remain largely unknown in most species, especially woody ornamentals [7, 8, 41].

*Rhododendron simsii* is a widely cultivated evergreen azalea characterized by rich flower‑color variation and extended flowering, and wild populations frequently occur in mountainous habitats with pronounced fluctuations in light conditions [42, 43]. Taxa from high-altitude or high-light environments often exhibit deeper or more vivid flower colors, suggesting a close link between anthocyanin metabolism, the light environment and flower-color phenotypes [34, 35]. Recent omics studies have begun to reveal the molecular basis of flower color variation in *Rhododendron* species, but the composition, expression patterns and light responsiveness of the GST family in this genus remain largely unexplored, and no systematic work has addressed the interplay between light-quality regulation, GST-mediated anthocyanin transport and flower color [11, 43, 44].

The availability of a chromosome-scale *R. simsii* genome now enables genome-wide dissection of its GST repertoire, and promoter cis-element prediction provides a basis for inferring potential light and stress responsiveness [42, 45, 46]. Building on these resources, we combined genome-wide bioinformatic analyzes in *R. simsii* with light-quality treatments in a pink-flowered cultivar of *Rhododendron hybridum* [34, 43]. We identified and characterized the *RsGST* gene family, including subfamily classification, chromosomal distribution, gene structures, conserved motifs and phylogenetic relationships, and analyzed promoter cis-elements with an emphasis on light-responsive motifs [47–49]. We then profiled tissue-, developmental-and light-quality-dependent expression of representative GST homologues in *R. hybridum*, together with dynamic changes in petal anthocyanin content, and integrated these datasets through a reciprocal best-hit framework [7, 15, 19]. Our goal was to pinpoint light-quality-responsive GST candidates, particularly within the Phi and Tau subfamilies, that may participate in vacuolar anthocyanin sequestration and flower color formation in *Rhododendron* [12, 15, 18].

## Methods

### Plant materials, growth conditions, and light-quality treatments

Five-year-old Pink Belgian azalea (Rhododendron hybridum; Yirun Horticulture, Ningbo, China) plants were acclimated in a controlled growth chamber (28 °C, 16 h light/8 h dark, 70–80% relative humidity, PPFD 1200–1250 µmol m⁻² s⁻¹) for 6 days. Fifteen uniform pots were randomly assigned to five light-quality treatments (*n* = 3 biological replicates per treatment): 365, 460, 660, and 730 nm LEDs, and full-spectrum white light (FSW, natural-light control). Light treatments were applied for 6 days, and flower buds and fully opened flowers were harvested at 0, 2, 4, and 6 days, snap-frozen in liquid nitrogen, and stored at − 80 °C until analysis. The chamber temperature was maintained at 28 °C throughout all light-quality treatments to minimize temperature-related confounding effects. In addition, *Nicotiana benthamiana* plants used for transient subcellular localization of *RhGSTF2* were grown in pots under standard greenhouse conditions at Zhejiang Wanli University until 4–5 weeks of age, and fully expanded leaves were selected for Agrobacterium-mediated transient expression.

### Identification of *RsGST* genes and cross-species orthology mapping (*R. simsii* → *R. hybridum*)

*Arabidopsis* GST proteins were retrieved from TAIR and the *R. simsii* genome/annotation from RPGD. Using the 55 AtGSTs as queries, we searched the *R. simsii* proteome with BLASTP (E-value ≤ 1 × 10⁻⁵). Candidate hits were validated by Pfam HMM and NCBI CDD; only sequences carrying canonical GST domains (e.g., GST_C_family/thioredoxin-like) were retained. Basic properties (length, MW, pI) were calculated with ExPASy ProtParam.

For orthology mapping to *R. hybridum*, the Rh protein set was formatted as a local database and queried with the 87 RsGST full-length proteins using TBtools (v2.0.41) local BLAST (BlastType=Guess, E-value ≤ 1 × 10⁻⁵). Hits were ranked by E-value, bit score, and alignment coverage; candidates with coverage ≥ 70% and identity ≥ 30% were kept, and the top-scoring hit (tie-break by higher coverage) was taken as the Rh counterpart (RBH confirmation where applicable). The Rs↔Rh mapping guided qPCR primer design and gene labels used throughout the study.

### Phylogenetic analysis and subfamily classification

Full-length GST amino acid sequences were aligned in MEGA (v11.0.13) using ClustalW. A neighbor-joining (NJ) phylogeny was then inferred with 1,000 bootstrap replicates, incorporating GSTs from *R. simsii* and *A. thaliana*. The resulting tree was visualized and color-coded by subfamily in Evolview (https://www.evolgenius.info/evolview-v2/).

### Chromosomal mapping, gene structure, and conserved motif analysis

Chromosomal coordinates and chromosome lengths were retrieved from the *R. simsii* genome annotation (GFF3). *RsGST* loci were mapped to chromosomes with TBtools (Gene Location Visualize from GTF/GFF), and exon–intron organizations were extracted from the same GFF3 and rendered in TBtools. Conserved protein motifs were identified using the MEME Suite (v5.5.2), with the search configured for 10 motifs and a motif width range of 6–50 amino acids (other parameters at default). The motif presence matrix was exported and integrated with the phylogeny and gene-structure panels for comparative visualization. Conserved domain assignments for all RsGST proteins were summarized in Supplementary Table S7. The consensus sequences of motifs 1–10 are provided in Supplementary Table S10.

### Cis-acting regulatory elements

For each *RsGST* gene, the 2-kb genomic sequence upstream of the translation start codon (ATG) was extracted from the *R. simsii* genome as the putative promoter region. Cis-acting regulatory elements were predicted using the PlantCARE database (http://bioinformatics.psb.ugent.be/webtools/plantcare/html/) with default settings. Detected elements were grouped into categories related to hormone responses, abiotic stress, light responsiveness, and development according to the PlantCARE annotation. The presence/absence matrix of elements across promoters was summarized by *RsGST* subfamily and visualized as heat maps in TBtools; element counts within 0–500 bp upstream of the transcription start site were additionally compared to highlight enriched light-responsive modules.

### Gene duplication and synteny analyzes

*RsGST* gene models were mapped to chromosomes with TBtools (Gene Location Visualize from GTF/GFF), and gene symbols were ordered/renamed by increasing physical position (5′→3′). Intra-genomic duplication was inferred using the TBtools one-step MCScanX (v1.0.0) workflow: all-vs-all BLASTP (E-value ≤ 1 × 10⁻⁵), collinearity detection by MCScanX, and duplicate classification. Tandem duplicates were defined as paralogous GSTs within a ≤ 200-kb window and separated by ≤ 1 intervening gene; segmental/WGD duplicates were assigned from collinear blocks. Inter-genomic synteny was visualized with Dual Synteny Plotter and Advanced Circos in TBtools using the same BLASTP settings, comparing GST regions between *R. simsii* and *A. thaliana*/*R. ovatum*. *R. ovatum* was chosen for inter-genomic synteny because a chromosome-level genome assembly with curated gene annotation is available, enabling robust genome-wide collinearity detection within the genus. *A. thaliana* was included as a distantly related outgroup for comparison. Genome resources for *R. simsii* and *R. ovatum* were obtained from public databases.

### RNA extraction and quantitative real-time PCR (qRT-PCR)

Total RNA was isolated using the YALEPIC^®^ Plant Total RNA Rapid Extraction Kit (PLUS) following the manufacturer’s protocol. RNA concentration and purity were assessed on a micro-volume spectrophotometer, and integrity was verified on 1% agarose gels. First-strand cDNA was synthesized with a Vazyme reverse-transcription kit, and qRT-PCR was performed with the corresponding Vazyme qPCR chemistry to profile tissue, developmental stage (S1–S4), and light-quality responses of target *RsGST* genes. Primer sequences are listed in Supplementary Table S5; ACTIN and EF1α served as reference genes. Relative transcript abundance was calculated using the 2^⁻ΔΔCt^ method.For tissue-specific expression (Fig. 6), heatmap values were gene-wise normalized for visualization by setting the lowest-expressing tissue for each gene as the calibrator (value = 1.0), and other tissues are presented as fold change relative to this calibrator (Supplementary Data 1). Each sample included three biological replicates, and each reaction was run in technical triplicate; melt-curve analysis confirmed single, specific amplicons. Petal developmental stages (S1-S4) were defined based on flower morphology, mainly the degree of flower opening and senescence status: S1, bud; S2, initial opening; S3, fully open; and S4, senescent (Supplementary Fig. S1). For light-quality treatments, flower buds (B) corresponded to stage S1 (closed, pre-anthesis buds), whereas fully open flowers (F) corresponded to stage S3 (anthesis stage with petals fully expanded), according to the morphological staging scheme (Supplementary Fig. S1).

### Subcellular localization prediction of *RsGST* proteins

The subcellular localization of RsGST proteins was predicted in silico using CELLO v2.5 (http://cello.life.nctu.edu.tw/) with the plant model and default parameters. Full-length amino acid sequences of the 87 RsGST proteins (Table S1) were submitted as input. For each protein, the primary subcellular compartment was defined as the location with the highest CELLO score. Scores for different compartments (extracellular space, plasma membrane, cytoplasm, endoplasmic reticulum, Golgi apparatus, lysosome, mitochondrion, chloroplast, peroxisome, vacuole and nucleus) were recorded for all RsGST proteins and summarized in Supplementary Table S6.

### Subcellular localization of *RhGST*F2 by transient expression

To examine the subcellular localization of a representative anthocyanin-related GST, the coding sequence of the *Rhododendron hybridum* orthologue of *RhGSTF2* was amplified without the stop codon and fused in-frame to GFP in the binary vector pCAMBIA1300-35 S::GFP. The empty pCAMBIA1300-35 S::GFP vector was used as a negative control. All constructs were confirmed by Sanger sequencing and introduced into *Agrobacterium tumefaciens* strain GV3101.

Agrobacterium cultures carrying the constructs were grown overnight, collected by centrifugation and resuspended in infiltration buffer (10 mM MgCl₂, 10 mM MES pH 5.6, 100 µM acetosyringone) to an OD₆₀₀ of 0.6. The suspensions were infiltrated into fully expanded leaves of 4–5-week-old Nicotiana benthamiana plants using a needleless syringe. After agroinfiltration, plants were kept in the dark for 12–16 h and then returned to normal conditions for 36–48 h before observation.

GFP fluorescence was observed with a Leica confocal laser scanning microscope (Leica Microsystems, Germany) using standard settings for GFP. Red autofluorescence from chlorophyll was recorded when needed. Fluorescence images were acquired using a confocal laser scanning microscope with a objective. GFP was excited at 488 nm and detected at 500–550 nm. The red channel was excited at 633 nm and detected at 650–750 nm. Images were acquired using identical settings for all samples within the same experiment.

### HPLC–DAD quantification of total anthocyanins in fully opened azalea flowers under short-term light-quality treatments

Petal extracts for HPLC–DAD were prepared by dispersing ~ 100 mg of frozen petal powder in methanol, sonicating for 10 min, and filtering the supernatant through a 0.22 μm membrane. Chromatography was performed on an Agilent Poroshell 120 EC-C18 column (4 μm, 4.6 × 150 mm) at 35 °C with a 5 µL injection, 1.0 mL min⁻¹ flow, and DAD monitoring at 520 nm. The mobile phases were A = 0.1% (v/v) trifluoroacetic acid in water and B = methanol; the gradient was 0–5 min, 95% A; 5–6 min, 95%→70% A; 6–25 min, 70% A; 25–26 min, 70%→15% A; 26–30 min, 15% A; 30–31 min, 15%→95% A; 31–35 min, 95% A (re-equilibration). Total anthocyanins were expressed as integrated peak area (AUC, mAU·min) by summing all peaks within 6–18 min on the 520 nm trace (*n* = 3 biological replicates per condition). Raw chromatograms were exported and plotted as stacked overlays in OriginPro (OriginPro 2022) without additional smoothing beyond instrument defaults. The raw HPLC–DAD chromatograms (A520) for all biological replicates and the corresponding peak-area integration Tables (6–18 min) used to calculate total anthocyanins are provided in Supplementary Data 4.

### Statistical analyzes and figure preparation

All statistics were performed in SPSS (IBM, Chicago, USA, v27.0.1). One-way ANOVA was used, and when significant, means were separated by Duncan’s multiple range test at α = 0.05 (two-tailed). For qRT-PCR datasets, statistical tests were performed on ΔCt values (technical triplicates averaged; *n* = 3 biological replicates) prior to fold-change transformation. Data are reported as mean ± SD of three biological replicates (technical triplicates averaged prior to analysis). Graphs were produced in GraphPad Prism (v9.5.1, GraphPad Software, San Diego, CA, USA) and figure panels were assembled in Adobe Illustrator (v28.6, Adobe Inc., San Jose, CA, USA).

## Results

### Genome-wide identification, physicochemical properties and phylogenetic classification of RsGST genes

Using BLAST searches with *Arabidopsis* GST protein sequences as queries, a total of 87 GST genes were identified in the *R. simsii* genome and designated *RsGST*1–*RsGST*87 according to their physical positions on the chromosomes. We then systematically analyzed several physicochemical properties of the corresponding RsGST proteins, including amino acid length, molecular weight (MW), theoretical isoelectric point (pI), instability index (II), aliphatic index (AI) and grand average of hydropathicity (GRAVY) (Table S1).

The RsGST proteins ranged from 91 to 826 amino acids (aa) in length, with an average of 251.6 aa and a median of 213 aa; approximately 67.8% (59 members) were between 100 and 300 aa. Their predicted MWs varied from 10.35 to 90.94 kDa, with an average of 28.30 kDa. The theoretical pI values ranged from 4.27 to 9.61, with an average of 6.33, and 74.7% (65 proteins) had pI values below 7, indicating that most RsGSTs are acidic proteins. GRAVY values were predominantly negative (73.6%), suggesting that the proteins are generally hydrophilic. The aliphatic index ranged from 73.56 to 119.58, with an average of 89.99. Based on the instability index (II ≤ 40 considered stable), 58.6% (51 proteins) were predicted to be stable. Overall, *RsGST* proteins are typically of moderate length, predominantly acidic, highly hydrophilic, and show generally good structural stability.

**Fig. 1:**
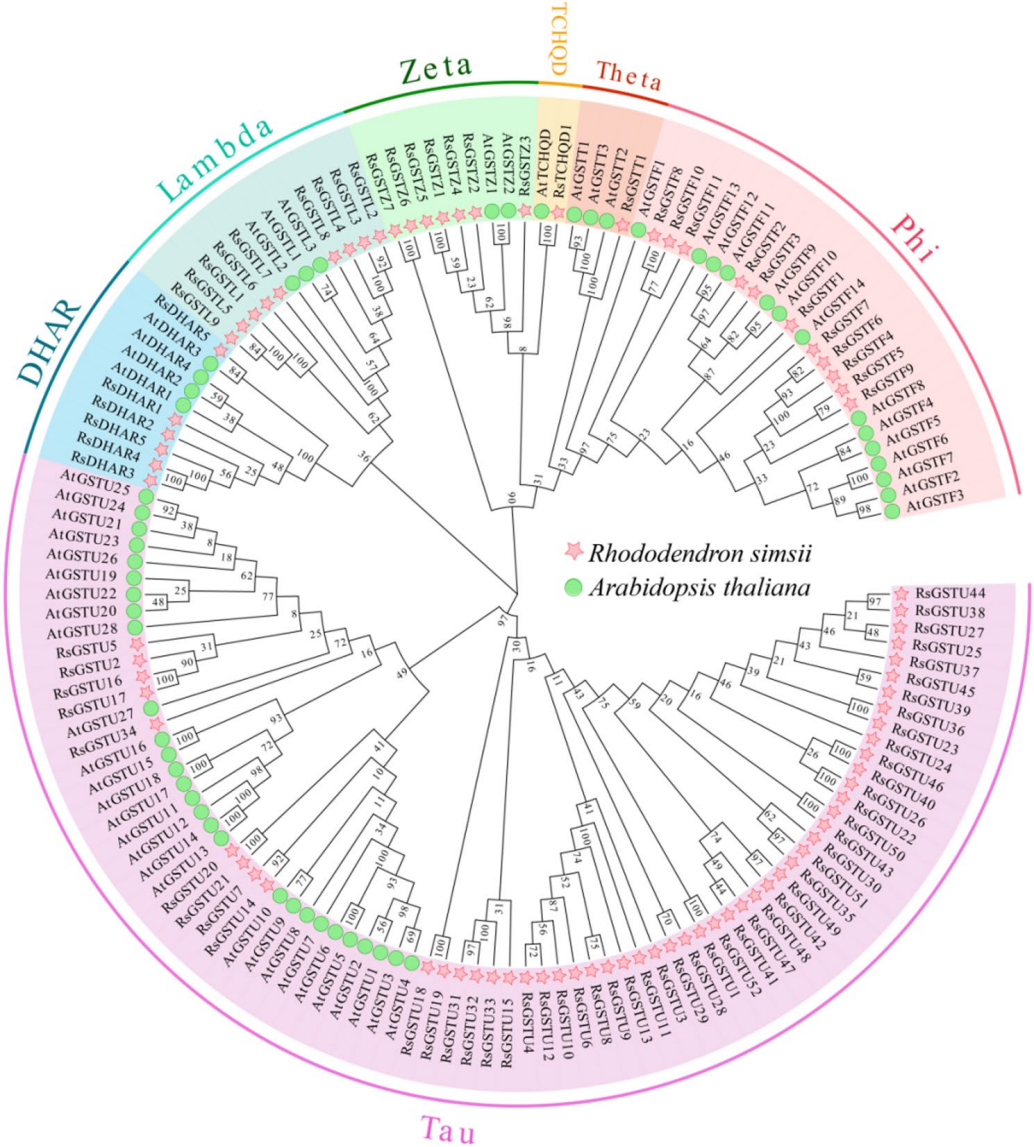
Phylogenetic relationships and subfamily classification of GSTs from *R. simsii* (Rs, *n* = 87) and *A. thaliana* (At, *n* = 55). The phylogenetic tree was reconstructed from full‑length GST protein sequences of *R. simsii* and *A. thaliana* using the neighbor‑joining method implemented in MEGA with 1,000 bootstrap replications. Colored sectors indicate GST subfamilies (Tau, Phi, Lambda, Zeta, DHAR, TCHQD, Theta). Green markers and red markers denote GSTs from *A. thaliana* and *R. simsii*, respectively

To clarify their evolutionary relationships and subfamily composition, a phylogenetic tree was constructed using the 87 RsGST proteins together with 55 AtGST proteins from *A. thaliana*. The combined tree resolved all GSTs into seven subfamilies: Tau, Phi, Lambda, Zeta, DHAR, TCHQD and Theta (Fig. 1). In *R. simsii*, Tau was the largest subfamily, comprising 52 members and accounting for 59.8% of the *RsGST* family, followed by the Phi subfamily with 11 members (12.6%). A similar pattern was observed in *A. thaliana*, in which Tau (28 members, 50.9%) and Phi (14 members, 25.5%) also represented the major subfamilies. The dominance of Tau and Phi in both species is consistent with the reported expansion pattern of plant GST families and may be closely linked to the functional diversification of these subfamilies in secondary metabolism.

Subfamily-level comparisons of physicochemical properties (Table S2) further revealed clear structural divergence among *RsGST* subfamilies. Tau and Phi members usually have moderate sequence lengths and relatively low instability indices, with Phi proteins being the most stable. By contrast, Lambda members tend to have longer amino acid sequences, higher molecular weights and increased instability, whereas DHAR proteins exhibit slightly higher (more basic) pI values and generally higher aliphatic indices. These differences indicate clear divergence in structural composition and stability among *RsGST* subfamilies, which may be closely related to their functional specialization. Taken together, genome-wide identification, physicochemical characterization and phylogenetic classification show that the *RsGST* gene family in *R. simsii* is dominated by Tau and Phi members and exhibits marked subfamily-specific structural divergence, providing a basis for subsequent analyzes of their specialized functions in secondary metabolism.

### Gene structure, domain architecture and conserved motif patterns of RsGST genes

**Fig. 2:**
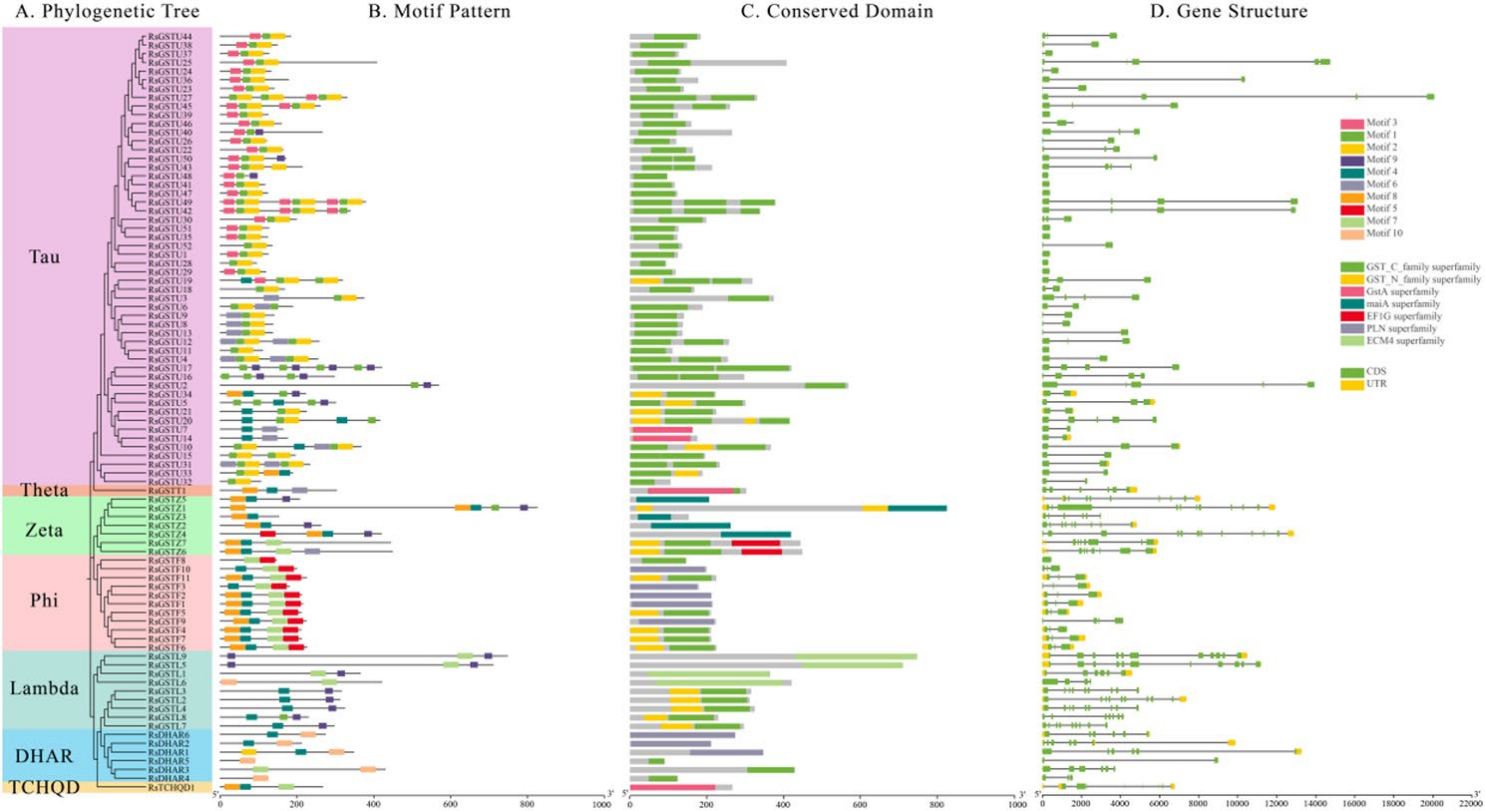
Phylogeny, conserved motifs, conserved domains, and gene structures of *RsGSTs. ***A** Neighbor‑joining phylogeny based on full‑length RsGST protein sequences, colored by subfamily. **B** Distribution of conserved motifs identified by MEME; colored blocks denote distinct motifs as indicated. **C** Conserved domain architecture predicted with NCBI CDD; colored blocks indicate domain types. **D** Exon–intron organization inferred from CDS–genome alignments; exons, introns, and UTRs are shown with standard symbols as indicated.The consensus sequences of motifs 1–10 are provided in Table S10

Using the GFF annotation of the *R.* simsii genome, we employed TBtools to generate an integrated view of the phylogenetic relationships, conserved motifs, conserved domains and exon–intron structures of the *RsGST* gene family (Fig. 2A–D). Genes within the same subfamily exhibited highly similar gene structures and motif compositions, whereas clear differences were observed among subfamilies. Specifically, members of the Tau and Phi subfamilies generally had shorter gene lengths and fewer introns; Zeta genes tended to be longer and contained more introns; Lambda genes were of intermediate to relatively long length; and both Theta and TCHQD were represented by single-copy genes. These structural characteristics were highly consistent with the phylogenetic groupings and were further supported by the patterns of conserved domains and motifs (Fig. 2B, C).

Most RsGST proteins contained typical GST-related domains. Among the 87 members, 77.0% (67 proteins) carried a C-terminal GST_C_family domain, and 25.3% (22 proteins) harbored an N-terminal Thioredoxin_like domain, with 19 proteins containing both domains. In addition, several proteins carried other domains, including PLN (8 members), maiA (5), ECM4 (4), GstA (4) and EF1G (2), reflecting considerable structural diversity within the family. At the subfamily level, GST_C_family was predominant in the Tau subfamily, present in 96.2% (50/52) of its members. In the Lambda subfamily, both Thioredoxin_like and GST_C_family domains were common, each occurring in 55.6% (5/9) of the members. In Zeta, the main domain was most frequent, found in 71.4% (5/7) of the proteins. In the Phi subfamily, 45.5% (5/11) of the proteins exhibited the classical dual-domain configuration (GST_C_family + Thioredoxin_like), whereas another 45.5% (5/11) contained only the PLN domain. In the DHAR subfamily, PLN and GST_C_family each accounted for 50% (3/6) of the domains. The single-copy Theta and TCHQD members possessed GST_C_family and GstA domains, respectively. Overall, the domain composition of each subfamily was highly concordant with its phylogenetic placement.

Using MEME, we identified 10 conserved motifs (motif-1 to motif-10; 21–41 aa in length) across the 87 RsGST proteins. The combination patterns of these motifs displayed clear subfamily specificity: Tau members were dominated by motif-1 and motif-2; Phi members consistently contained motif-4 and motif-6, with most also harboring motif-3 and motif-7; Zeta members generally carried motif-3 and motif-7, with some also containing motif-8; Lambda proteins were characterized by motif-8, frequently in combination with motif-3 and motif-6; and DHAR members were mainly associated with motif-9. The single-copy Theta and TCHQD proteins showed motif combinations of motif-5/7/3 and motif-3/6/7, respectively (Fig. 2B). In terms of positional distribution, motif-7 and motif-2 were predominantly located at the N terminus (84.2% and 71.9% of occurrences, respectively); motif-6 and motif-1 were mostly found in the central region (76.5% and 51.8%); motif-3 and motif-5 spanned the N-terminal to central regions (54.5% and 43.8%); whereas motif-8 and motif-4 were strongly biased towards the C terminus (76.9% and 91.7%, respectively). This pattern is consistent with the functional partitioning of GST proteins into an N-terminal thioredoxin-like domain (G-site) and a C-terminal hydrophobic substrate-binding region (H-site).

Taken together, the *RsGST* gene family exhibits a clear pattern of being conserved within but divergent between subfamilies in terms of exon–intron organization, domain architecture and motif composition. This modular structural organization is highly consistent with the phylogenetic subfamily divisions and suggests that subfamily‑specific structural features have been established during evolution in parallel with functional diversification.

### Chromosomal distribution and gene duplication of *RsGST* genes

**Fig. 3:**
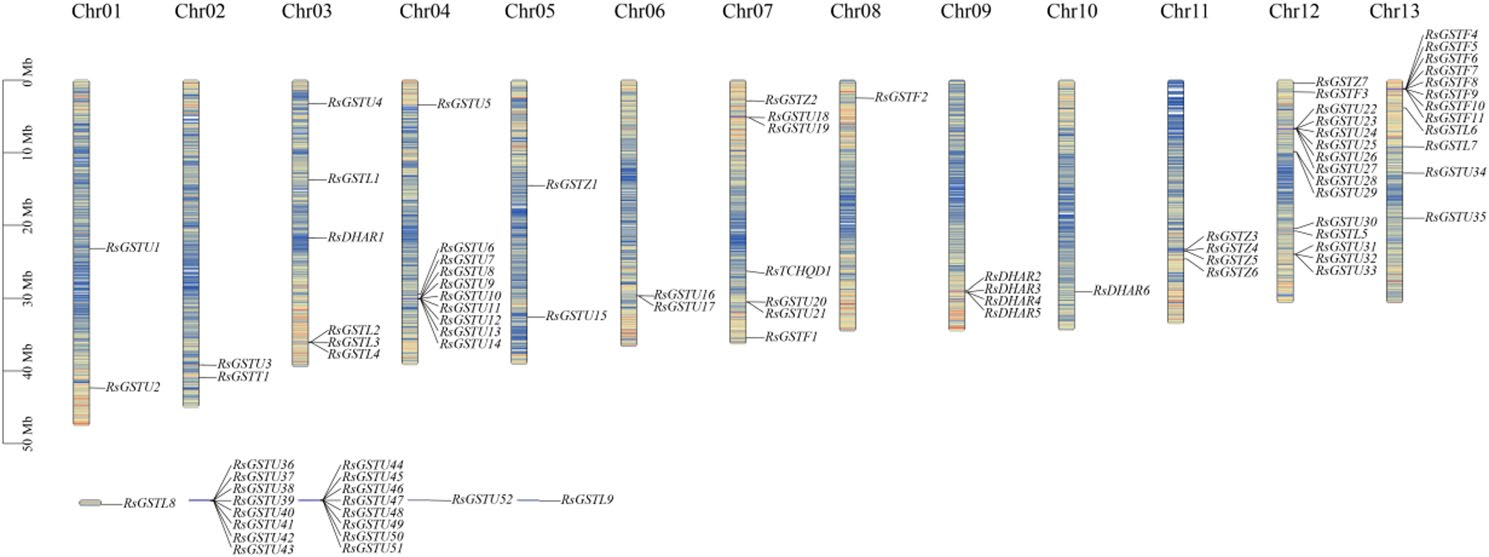
Chromosomal distribution of *RsGST* genes in *R. simsii. *Physical positions were obtained from the genome annotation and visualized in TBtools. Vertical bars represent the 13 chromosomes of *R. simsii*, labeled at the top and drawn to scale; the megabase scale is shown on the left. Genes not anchored to chromosomes are placed at the bottom. In total, 68 *RsGSTs* map to the 13 chromosomes and 19 reside on unanchored scaffolds(Table S8)

In the *R. simsii* genome, the 87 identified *RsGST* genes were systematically named according to their subfamily assignment and physical positions as *RsGSTF1–F11*,* RsGSTT1*,* RsTCHQD1*,* RsGSTZ1–Z7*,* RsGSTL1–L9*,* RsDHAR1–D6 and RsGSTU1–U52* (Fig. 3). Among these, 68 genes were successfully anchored to 13 chromosomes, whereas the remaining 19 genes could not yet be assigned to specific chromosomes. The mapped *RsGST* genes showed an uneven distribution across chromosomes. Chromosomes 12 and 13 contained the largest numbers of *RsGSTs*, with 15 and 12 genes, respectively, together accounting for 31.0% (27/87) of the family, while only a single *RsGST* gene was detected on chromosome 10. At the subfamily level, the Tau subfamily was not only the largest but also the most widely distributed, forming obvious gene clusters on chromosomes 4 and 12, where 22 Tau members were concentrated, representing 25.3% of all *RsGST* genes.

Using genome annotation and chromosomal mapping information, a total of 87 *RsGST* genes were identified in the *R. simsii* genome. Among these, 68 genes were unambiguously anchored to 13 chromosomes, whereas 19 genes remained unassigned to specific chromosomes (Fig. 3). The chromosomally mapped *RsGST* genes were unevenly distributed, with obvious gene clusters observed on several chromosomes (Fig. 3).

**Fig. 4:**
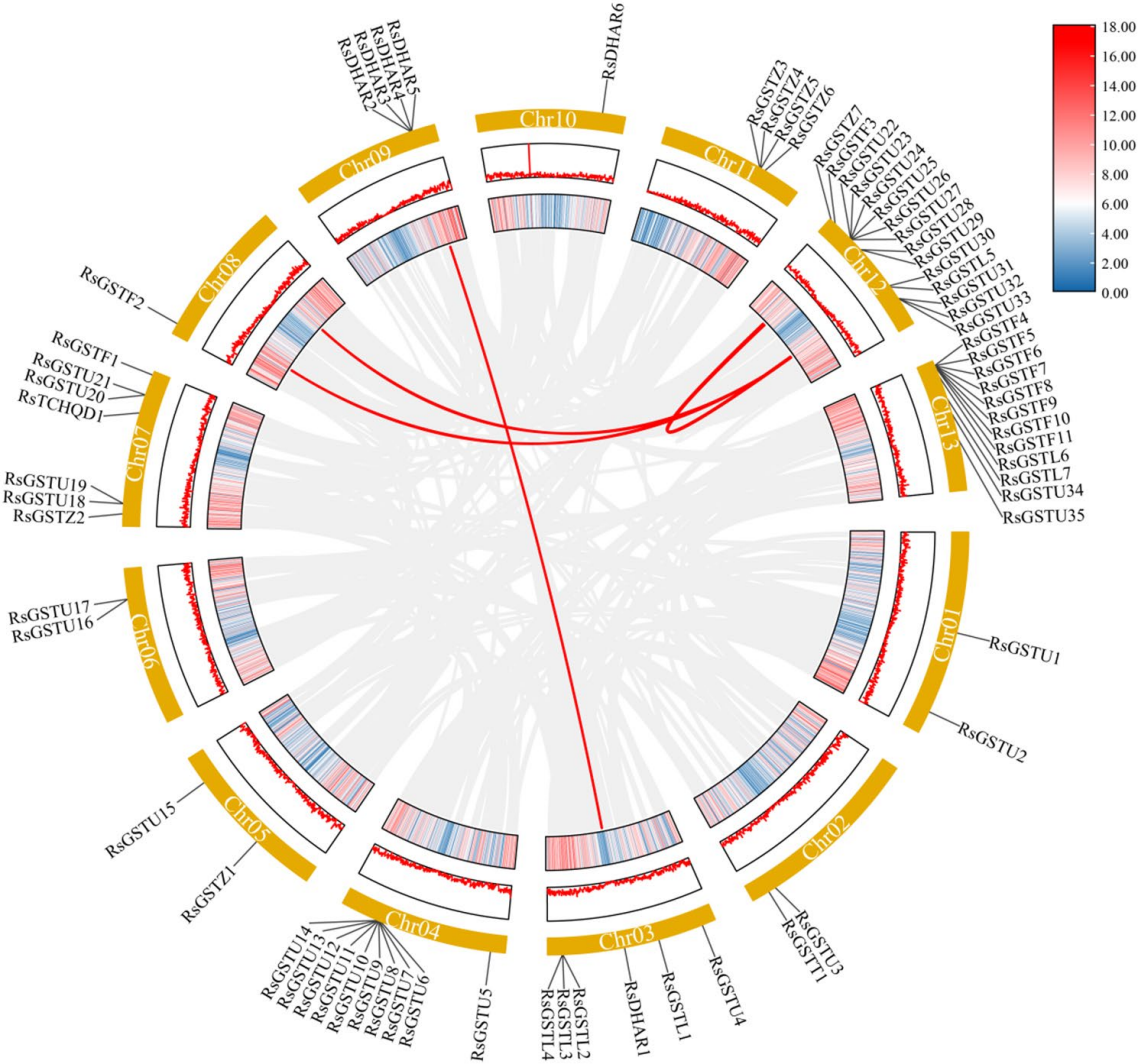
Intra‑genomic synteny of GST genes in *R. simsii. *Circos diagram depicting intra‑genomic synteny across the 13 chromosomes of *R. simsii* (CM024953.1–CM024965.1). Gray ribbons connect genome‑wide collinear gene pairs. Red lines highlight collinear blocks containing *RsGST* genes, with colors indicating GST subfamilies. Chromosome numbers and lengths (Mb) are shown in the outer ring; inner ticks mark *RsGST* loci

Synteny analysis further revealed multiple interchromosomal collinear blocks involving RsGST loci. In combination with the presence of local tandem clusters, these results indicate that expansion of the RsGST family has occurred through multiple duplication modes. Segmental/WGD-derived collinearity and chromosomal rearrangements contributed to the retention of a subset of loci in conserved blocks, whereas many Tau/Phi members likely expanded via tandem/proximal and other lineage-specific duplications that typically do not reside in detectable collinear blocks under MCScanX criteria (Fig. 4). According to the MCScanX duplication-type classifier, the 87 RsGST genes were categorized as 41 tandem, 13 proximal, 21 dispersed, 10 WGD/segmental, and 2 singleton genes (Table S9).

### Inter-species synteny and selection pressure on *RsGST* genes

**Fig. 5:**
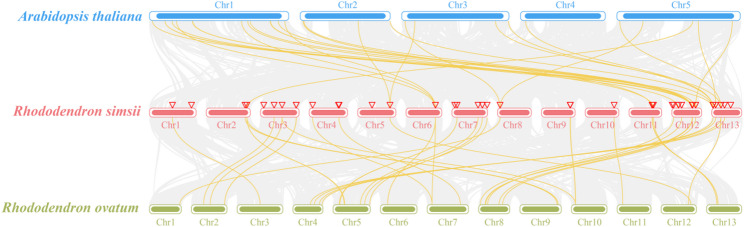
Inter‑genomic synteny among *A. thaliana*, *R. simsii*, and *R. ovatum. *Synteny map among *A. thaliana* (Chr1–Chr5), *R. simsii* (Chr1–Chr13), and *R. ovatum* (Chr1–Chr13). Gray curves represent genome‑wide collinear gene pairs, whereas yellow curves highlight collinear blocks containing GST genes. Chromosomes are drawn to scale and labeled; GST loci are indicated along each chromosome. Species are color‑coded (*A. thaliana*, blue; *R. simsii*, red; *R. ovatum*, green)

To assess conservation of GST loci within *Rhododendron*, we performed inter-species collinearity comparisons between *R. simsii* and *R. ovatum*, a closely related species with a publicly available chromosome-level reference genome and annotation; *A. thaliana* was included as a distantly related outgroup reference (Fig. 5). In the comparison between *R. simsii* and *R. ovatum*, 14 pairs of GST orthologues were identified, distributed among the Tau (5 pairs), Lambda (3 pairs), DHAR (2 pairs), Zeta (2 pairs), Phi (1 pair) and TCHQD (1 pair) subfamilies. In the *R. simsii* genome, these orthologous genes were mainly located on chromosomes 3 (3 pairs), 7 (3 pairs), 11 (2 pairs), 12 (2 pairs) and 13 (2 pairs), with chromosomes 4 and 10 each harboring one pair; representative genes included *RsDHAR1*,* RsGSTZ3*,* RsGSTL1*,* RsGSTU28*,* RsGSTU5* and *RsTCHQD1*. By contrast, the comparison between *R. simsii* and *A. thaliana* detected only scattered interchromosomal syntenic links involving 14 *RsGST* loci and forming 28 pairwise connections, but no continuous collinear blocks. This pattern reflects a pronounced erosion of collinearity caused by long-term divergence between these distantly related species.

Overall, the GST syntenic relationships between *R. simsii* and *R. ovatum* are more complete, with clearly defined collinear blocks, whereas only fragmented interchromosomal correspondences remain between *R. simsii* and *A. thaliana*, lacking extended blocks. This indicates that the conserved structural modules of the GST family have been largely maintained within the genus *Rhododendron*. In addition, many members of the Tau subfamily did not form clear orthologous pairs in the inter-species comparison, which is consistent with their evolutionary tendency to undergo tandem duplication and lineage-specific expansion.

On the basis of the synteny results, we further estimated selective constraints on *RsGST* genes by calculating Ka/Ks ratios for duplicated gene pairs within *R. simsii* and for orthologous pairs between *R. simsii* and *A. thaliana* (Table S3). In the intraspecific comparison, four valid paralogous gene pairs were obtained, with Ka/Ks values ranging from 0.216 to 0.354 and a median of 0.259 (median Ka = 0.302, median Ks = 1.093). All ratios were < 1, indicating that these duplicated genes have been subject to strong purifying selection; among them, *RsDHAR1–RsDHAR4* showed the highest Ka/Ks value (0.354). In the interspecific comparison, 16 orthologous pairs between *R. simsii* and *A. thaliana* were retained, with Ka/Ks values ranging from 0.043 to 0.397 and a median of 0.126 (median Ka = 0.334, median Ks = 2.285), again with all values < 1. The highest ratio was observed for *RsGSTU31–AT5G62480.1* (0.397). Using a synonymous substitution rate for dicotyledonous plants of λ = 6.5 × 10⁻⁹ substitutions·site⁻¹·year⁻¹, the median Ks values correspond to an estimated divergence time of ~ 176 Ma for the *R. simsii*–*A. thaliana* orthologs and ~ 84 Ma for the intraspecific duplication events.

Taken together, these results indicate that the *RsGST* gene family has been predominantly shaped by long-term purifying selection at both the intra- and interspecific levels, with only a small subset of loci showing signs of slight functional divergence.

### Tissue and developmental stage–specific expression of *RsGST* genes

**Fig. 6:**
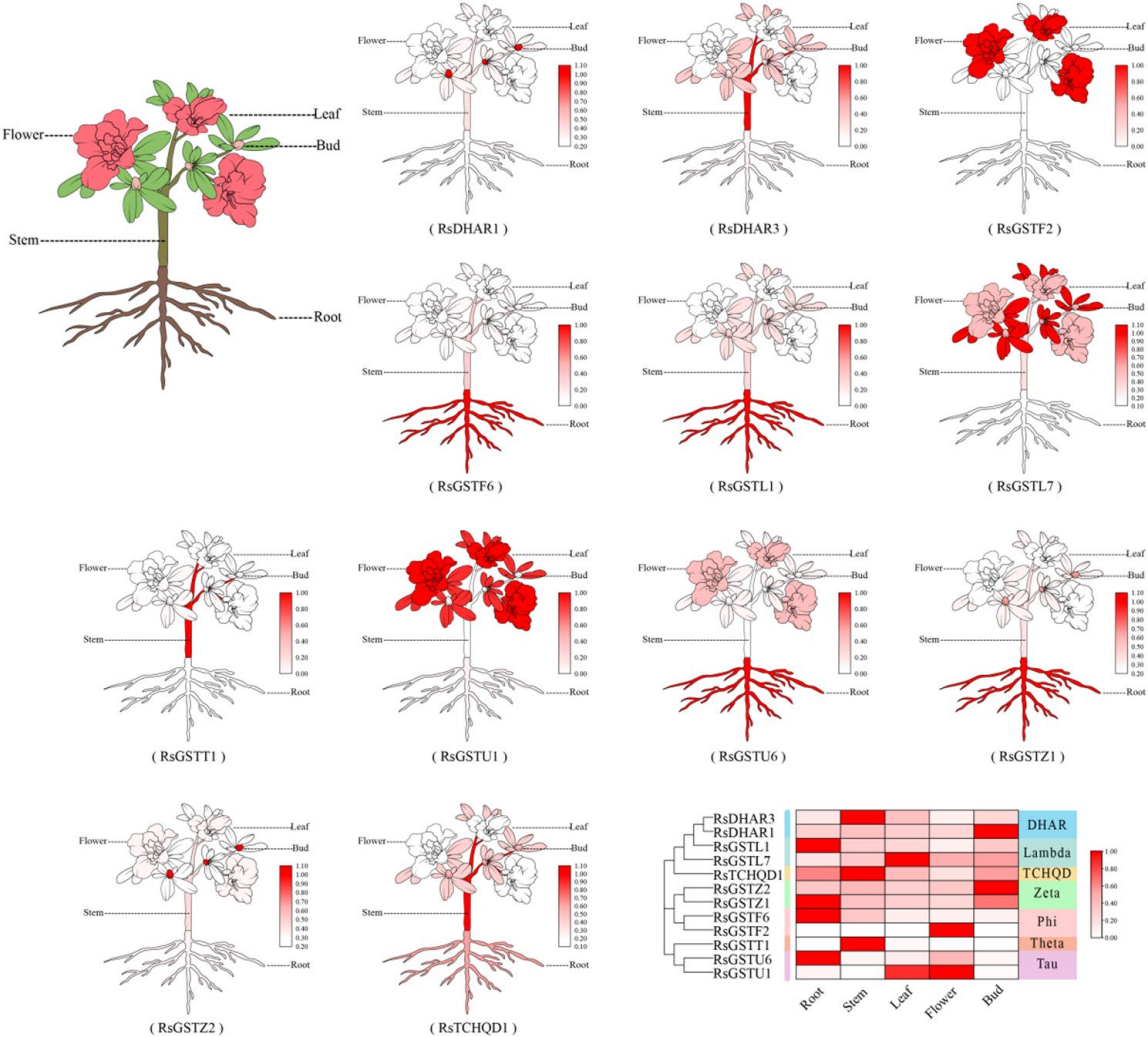
Tissue-specific expression of *RhGST* orthologs in *Rhododendron hybridum. *The left schematic indicates the five sampled tissues. For each of the 12 genes, red intensity marks normalized relative expression (white = low, deep red = high). The heatmap summarizes the expression matrix across Root, Stem, Leaf, Flower and Bud with hierarchical clustering; the sidebar annotates GST subfamilies(Supplementary Data 1). For each gene, the tissue with the lowest mean expression was used as the calibrator (set to 1.0) for gene-wise normalization

Tissue expression profiling revealed pronounced tissue specificity of these 12 genes in roots, stems, leaves, flower buds and fully open flowers, allowing them to be grouped into three categories: (i) root/stem-preferential genes (e.g., *RsGSTF6*,* RsGSTL1*,* RsGSTU6* and *RsGSTZ1*), which showed higher expression in roots or stems and may participate in detoxification and antioxidant processes; (ii) reproductive organ–preferential genes (e.g., *RsGSTF2*,* RsGSTL7* and *RsGSTU1*), which were specifically and highly expressed in flowers or flower buds, suggesting roles in flower color formation; and (iii) stem-biased genes (e.g., *RsGSTT1* and *RsDHAR3*), which were predominantly expressed in stems and may be involved in stem-specific redox regulation. Most genes showed relatively low expression in leaves, although *RsGSTU1*,* RsGSTT1* and *RsGSTL7* also accumulated to medium or high levels in leaf tissue (Fig. 6).

**Fig. 7:**
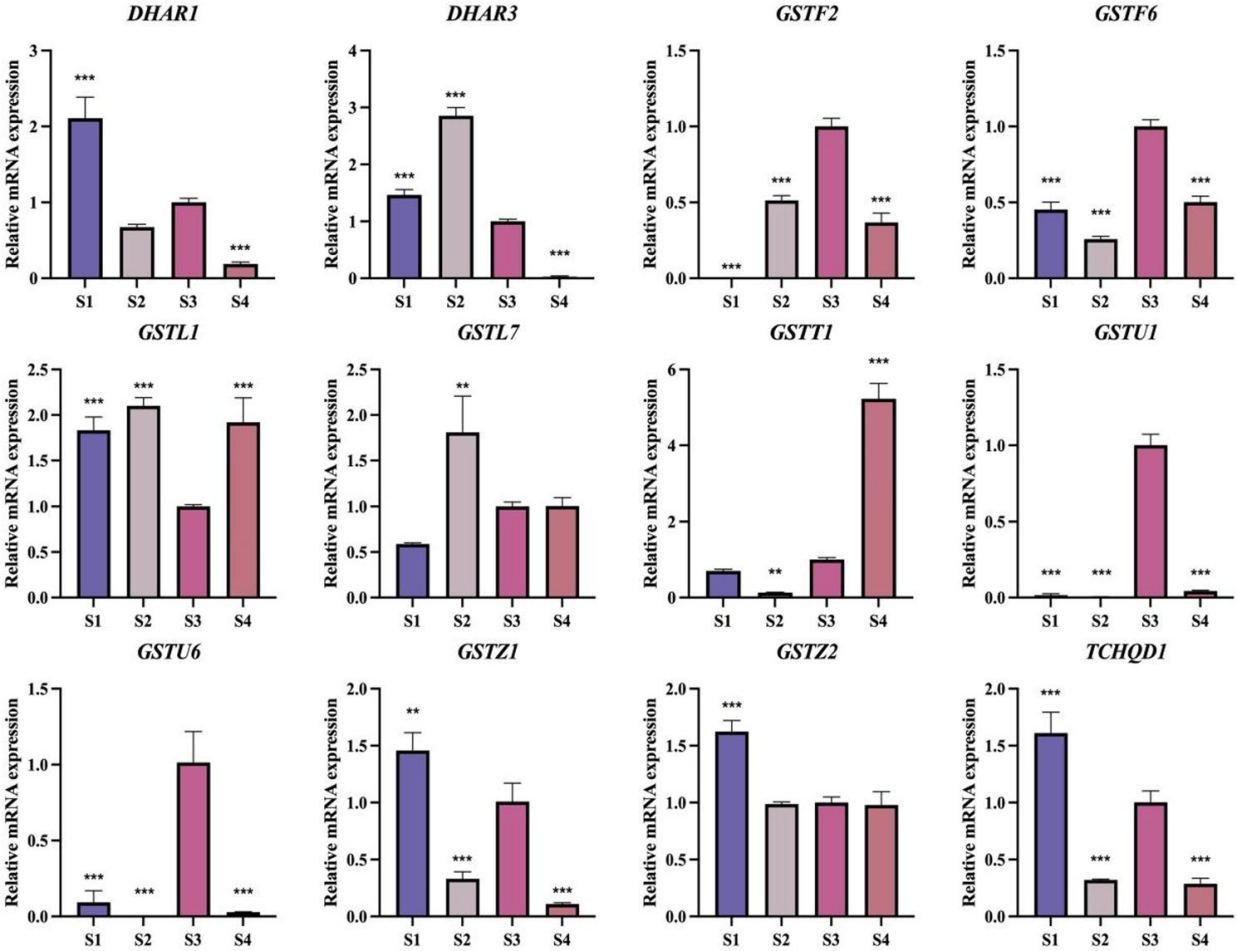
Stage-specific expression of RhGSTs during flower development. Relative transcript levels of 12 representative RhGST genes were quantified by qRT-PCR in *R. hybridum* flower petals at four developmental stages (S1–S4)(Supplementary Data 2). Stage definitions (S1-S4) are provided in Supplementary Fig. S1. Asterisks indicate significant differences compared with S3 at the same time point (*P* < 0.05, *P* < 0.01, *P* < 0.001)

Across the four defined stages of petal development (S1–S4), the representative *RsGST* homologues exhibited distinct temporal expression dynamics (Fig. 7). Most genes were expressed at low levels during the bud stage (S1), whereas several, such as *RsGSTF2*,* RsGSTU1* and *RsGSTU6*, were markedly upregulated as flowers opened (S2–S3), implying potential roles in anthocyanin transport and accumulation. By contrast, genes such as *RsGSTZ1* and *RsTCHQD1* showed decreased expression from S3 to S4, indicating that their functions may be confined to earlier stages of flower development. Notably, *RsGSTL1* and *RsGSTT1* maintained relatively high expression even at the senescent stage (S4), suggesting possible roles in preserving floral organ stability or mediating antioxidant regulation during late development. The morphological criteria used to define stages S1-S4 are shown in Supplementary Fig. S1.

Collectively, these *RsGST* homologues display highly dynamic, tissue- and stage-specific expression patterns, highlighting the functional diversity of the GST family in flower color formation, stress responses and metabolic regulation, and further supporting their important regulatory roles during *Rhododendron* flower development.

### Cis-acting elements in the promoters of *RsGST* genes

**Fig. 8:**
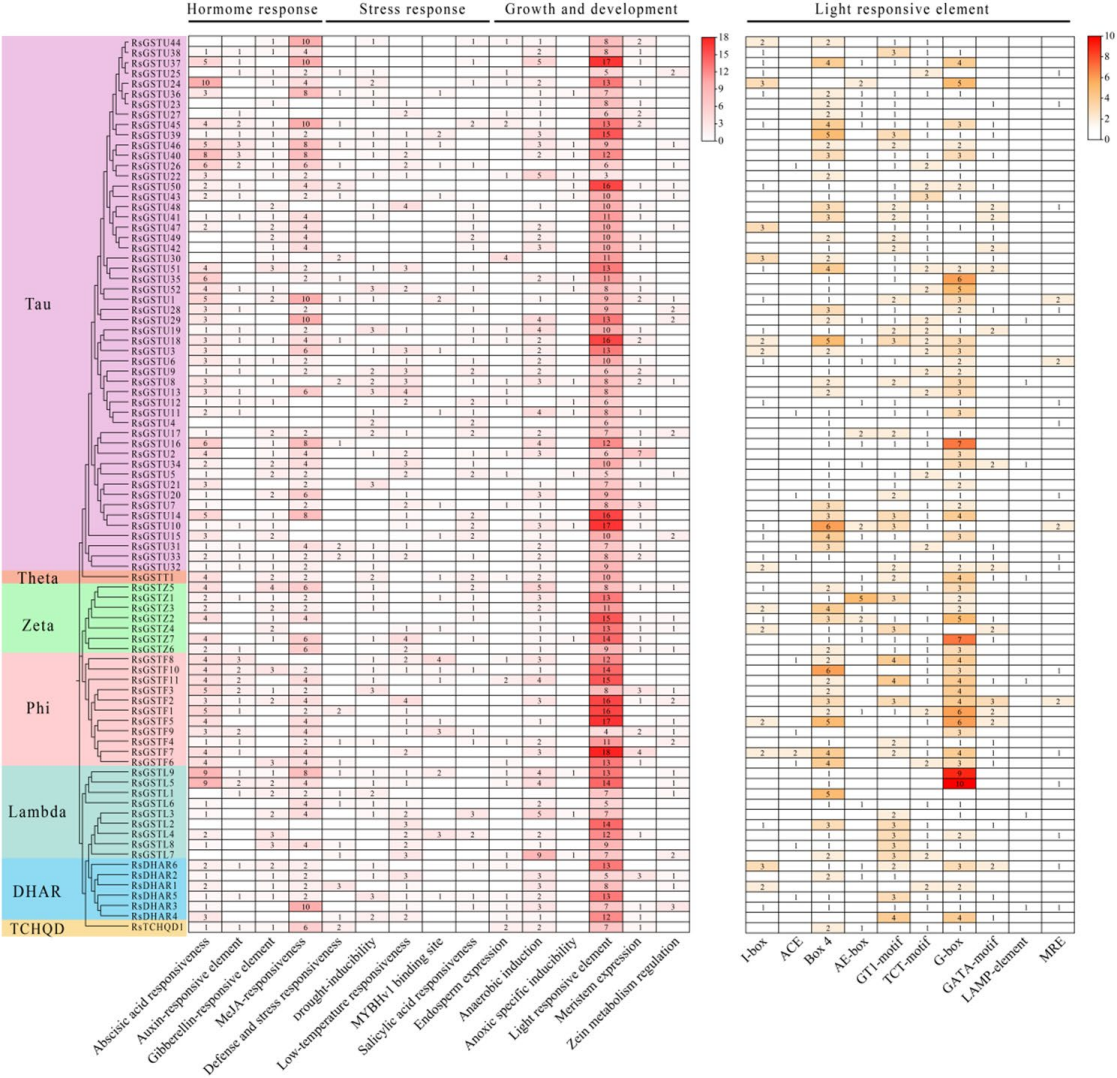
Cis‑acting elements in the 2‑kb upstream promoters of *RsGST* genes and light‑responsive modules. **a** Overview of cis‑regulatory elements associated with hormone responses, abiotic stress and development. **b** Gene‑by‑element heat map of light‑responsive elements; G‑box (CACGTG) and ACGT‑family elements (ACE, Z‑box, T/G‑box) are enriched, followed by I‑box, GT1‑motif, GATA‑motif, TCT‑motif, Box 4, AE‑box, LAMP‑element, and MRE. Elements are enriched near the transcription start site (0–500 bp). Color intensity reflects element counts; subfamilies are indicated in side bars

Based on cis-acting element analysis of the 2 kb upstream promoter regions of the 87 *RsGST* genes, we systematically identified elements associated with hormone responses, stress responses, light responsiveness and growth and development, and visualized their distribution together with the phylogenetic relationships in a heatmap (Fig. 8). For hormone-responsive elements, abscisic acid-responsive ABRE, methyl jasmonate-responsive MeJA elements and gibberellin-responsive GA elements showed the highest coverage, being present in 85.1% (74/87), 80.5% (70/87) and 65.5% (57/87) of promoters, respectively, whereas auxin- and salicylic acid-related elements were detected in 48.3% (42/87) and 49.4% (43/87) of promoters. Among the 74 promoters with complete upstream sequence annotation, light-responsive elements were generally enriched. On average, each promoter contained 8.38 core light-responsive elements, with Box 4 and G-box showing the highest occurrence frequencies, present in 89.2% (66/74) and 81.1% (60/74) of promoters, respectively. GT1-motif and TCT-motif were also common (74.3%, 55/74 and 67.6%, 50/74), whereas A-box, GATA-motif and I-box occurred at relatively low frequencies, and ACE and LAMP-element were confined to a small subset of promoters. Among stress-related elements, STRE (81.1%) and ARE (74.3%) were the most prevalent, while LTR, MBS and WUN-motif were also widely distributed, suggesting a broad potential involvement of *RsGST* genes in abiotic stress responses.

Overall, the promoter regions of *RsGST* genes exhibit a regulatory landscape dominated by light- and hormone-responsive elements, superimposed on a typical stress-responsive signature. Subfamily-level comparisons of the total number of light-responsive elements showed that Phi members harbored on average 11.36 such elements per promoter, significantly more than Zeta (10.00), Tau (7.65) and Lambda (7.00) members (Fig. 8). This difference points to possible functional partitioning among subfamilies in light perception and signaling, and provides a cis-regulatory basis for interpreting their divergent expression patterns during light-induced anthocyanin accumulation.

### Light-quality–dependent expression patterns of RhGSTs

To examine the light-quality responsiveness of GST genes, two-year-old *R. hybridum* plants were exposed to 365 nm UV-A, 460 nm blue light, 660 nm red light, 730 nm far-red light and full-spectrum white light (FSW), and the expression dynamics of 12 *RsGST* orthologues (hereafter collectively referred to as *RhGSTs*) were analyzed by qRT–PCR. The results showed pronounced light-, organ- and time-specific expression patterns. In flower buds, UV-A and blue light triggered a rapid and strong induction, with median expression levels reaching 6.36- and 7.00-fold those of the FSW control on day 2, respectively. By contrast, 730 nm far-red light caused a delayed response, with 9 of the 12 *RhGSTs* reaching their peak expression only on day 6. In fully open flowers, the genes were more sensitive to red and far-red light, with median expression levels increasing 6.05- and 6.49-fold on day 2, respectively, indicating an earlier overall response than in buds and a distinct shift in spectral preference. These findings reveal a spatiotemporally specific regulatory mechanism whereby different light qualities modulate *RhGST* expression during floral development and pigmentation.

**Fig. 9:**
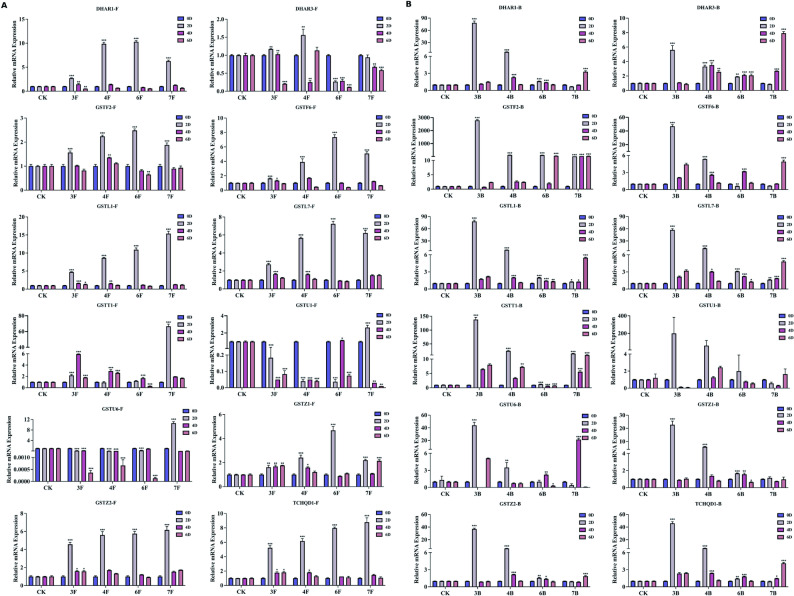
Spatiotemporal Expression Patterns of *RhGSTs* under Different Light-Quality Treatments. **A** Fully open flowers (F; stage S3) and **B** flower buds (B; stage S1) of R. hybridum were exposed to monochromatic LEDs at 365, 460, 660, and 730 nm or to the control condition under full-spectrum white light (FSW, CK) for 0, 2, 4, and 6 days. Relative transcript levels of 12 representative genes were quantified by qRT-PCR (Supplementary Data 3). Stage definitions are provided in Supplementary Fig. S1. Abbreviations: CK (FSW), full-spectrum white light control; 3, 4, 6, and 7 denote LED wavelengths of 365, 460, 660, and 730 nm, respectively; F, fully open flower; B, flower bud. Accordingly, 3F/4F/6F/7F indicate open flowers exposed to 365/460/660/730 nm LEDs, and 3B/4B/6B/7B indicate buds exposed to the same wavelengths. Asterisks indicate significant differences compared with CK at the same time point (*P* < 0.05, *P* < 0.01, *P* < 0.001)

### Total anthocyanin content in fully open flowers under different light-quality treatments

**Fig. 10:**
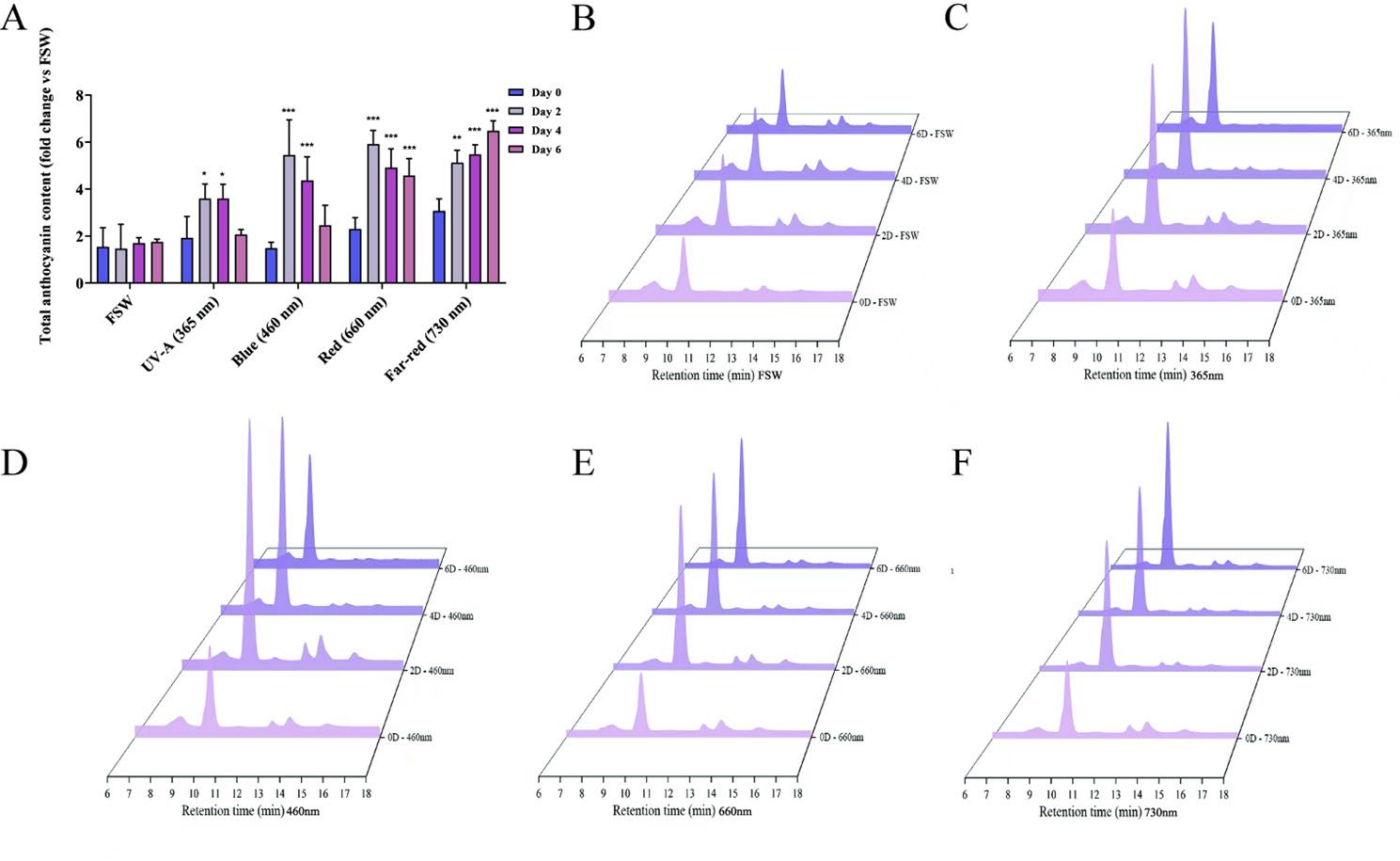
Total anthocyanin content of fully opened flowers under short-term light-quality treatments (HPLC-DAD, 520 nm). Panela(**A**) represent total anthocyanin content (fold change vs. FSW), Panels (**B**-**F**) represent the treatments with full-spectrum white light (FSW), 365, 460, 660, 730 nm, respectively. For each panel, the samples from 0, 2, 4, and 6 days of treatment are shown from bottom to top. The x-axis denotes retention time (min), with the 6–18 min interval used for total anthocyanin peak area calculation, and the y-axis represents absorbance (mAU)(Supplementary Data 4). Asterisks indicate significant differences compared with FSW at the same time point (*P* < 0.05, *P* < 0.01, *P* < 0.001)

To dissect the regulatory effects of light quality on anthocyanin accumulation, total anthocyanin contents in petal samples collected after the different light treatments were quantified by HPLC–DAD. The underlying chromatograms and quantification source data are available in Supplementary Data 4. The results showed that all light-quality treatments promoted anthocyanin accumulation, with the most pronounced increases occurring between 2 and 4 days after treatment. Specifically, on day 2, total anthocyanin contents in the 365 nm, 460 nm, 660 nm and 730 nm groups were 2.46-, 3.74-, 4.06- and 3.51-fold those of the full-spectrum white light (FSW) control, respectively. By day 4, treated plants still maintained 2.11- to 3.22-fold higher levels than the control. By day 6, anthocyanin contents in the 365 nm and 460 nm treatments had declined to 1.18- and 1.40-fold of the control, whereas the 660 nm and 730 nm treatments continued to sustain high levels at 2.62- and 3.71-fold, respectively (Figure 10). Collectively, these data indicate that although UV-A and blue light can rapidly induce anthocyanin biosynthesis, red and far-red light are more effective in maintaining long-term anthocyanin accumulation.

### Subcellular localization of candidate GST proteins

**Fig. 11:**
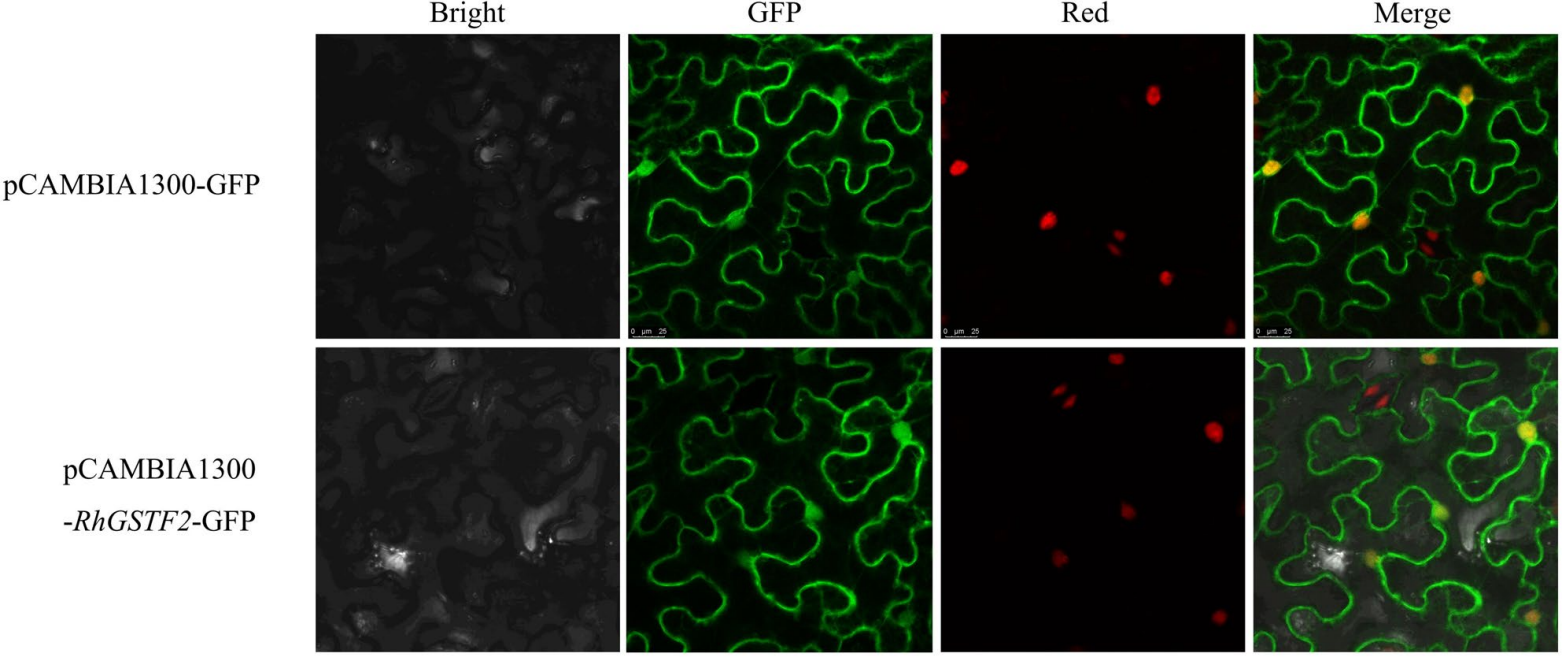
Subcellular localization of RhGSTF2–GFP in Nicotiana benthamiana leaf epidermal cells. GFP fluorescence (green) was recorded using Ex 488 nm / Em 500–550 nm, and the red channel was recorded using Ex 633 nm / Em 650–750 nm. The coding sequence of RhGSTF2 was fused in-frame to GFP under the control of the CaMV 35S promoter (bottom row), and the empty pCAMBIA1300–GFP vector was used as a control (top row). Green channel shows GFP fluorescence; red channel shows red autofluorescence; merged images are shown in the right column

To experimentally determine where *RhGSTF2* acts in the cell, its coding sequence without the stop codon was fused in-frame to GFP under the control of the CaMV 35 S promoter and transiently expressed in Nicotiana benthamiana leaf epidermal cells. In cells expressing 35 S::*RhGSTF2*–GFP, strong green fluorescence was observed outlining the characteristic jigsaw-shaped contours of epidermal cells and in round structures corresponding to nuclei, whereas little diffuse cytosolic signal was detected (Fig. 11). By contrast, the free GFP control driven by the same promoter showed a uniform distribution throughout the cytoplasm and nuclei and only weak signal along the cell periphery. These observations indicate that *RhGSTF2* is associated with the plasma membrane and accumulates in the nucleus, suggesting a dual localization pattern.

Notably, the in silico prediction from CELLO v2.5 classified *RhGSTF2* mainly as a cytoplasmic protein, with moderate nuclear and low plasma membrane scores (Table S6). The difference between the predicted cytoplasmic localization and the experimentally observed plasma membrane/nuclear distribution highlights the limitations of purely computational approaches and suggests that *RhGSTF2* may participate in processes at the cell surface and in the nucleus in addition to its putative cytosolic functions. *RhGSTF2* was chosen as a representative Phi-class candidate due to its strong flower-preferential and light-responsive expression; *RhGSTL7* and *RhGSTU1* will be examined in future work.

## Discussion

The GST gene family in *Rhododendron simsii* shows a mixture of conserved and lineage‑specific features when compared with other plant species [4, 50, 51]. In terms of family size and subfamily composition, the total number of GSTs and the numerical dominance of Tau and Phi members broadly resemble patterns reported in *Arabidopsis*, rice, wheat, grape and other crops, where expansion of these two subfamilies has repeatedly been associated with detoxification, stress adaptation and the handling of secondary metabolites, including anthocyanins [1, 2, 5]. At the same time, *R. simsii* harbors an unusually high proportion of Tau genes and several Lambda, Zeta and DHAR members with extended gene length, complex domain architecture and predicted targeting to organelles such as chloroplasts and mitochondria [3]. Together with the “conserved‑within, divergent‑between” pattern of exon‑intron organization and motif composition across subfamilies, these features suggest that *Rhododendron* has retained the canonical anthocyanin‑related GST modules while also evolving structural innovations that may help this woody ornamental cope with heterogeneous mountain habitats, fluctuating light conditions and correspondingly diverse flower‑color phenotypes [4, 43].

The transcriptional and cis‑regulatory landscapes of GST genes further underscore both conservation and divergence in their control across species [6, 7, 29]. In *Rhododendron*, representative GSTs can be broadly grouped into root/stem‑dominant, reproductive‑organ‑dominant and stem‑biased categories, echoing the functional partitioning seen in other plants between detoxification- and stress‑related GSTs in vegetative tissues and pigment‑associated GSTs in flowers and fruits [16, 19]. However, the precise deployment of these expression modules, and their sensitivity to developmental stage and light, differ from species to species, reflecting lineage‑specific tuning of regulatory networks [7, 34, 35]. Promoter analysis points to a regulatory framework in which light‑responsive and hormone‑responsive elements form the core of the GST cis‑landscape, superimposed on a background of general stress‑responsive motifs [5, 33, 46]. Consistent with this interpretation, light-responsive elements were generally enriched across the promoters with complete upstream annotation, with Box 4 and the G-box showing the highest occurrence frequencies (89.2% and 81.1%, respectively), and GT1-motif and TCT-motif also being common. Moreover, multiple ACGT-core modules (e.g., G-box/ACE/Z-box/T/G-box) were enriched and tended to cluster near the transcription start site (0–500 bp), a positional feature that is often associated with strong responsiveness to upstream signaling inputs.These cis-features provide a plausible mechanistic bridge between photoreceptor-driven light signaling and the observed spectral and stage specificity of GST transcription: for instance, our qRT-PCR results indicate that buds show stronger early induction under UV-A/blue, whereas fully open flowers respond more strongly and earlier to red/far-red light, implying that distinct light-quality pathways converge on overlapping sets of GST promoters but with developmentally shifted sensitivity. In parallel, hormone-responsive elements were also widespread (e.g., ABRE and MeJA-responsive modules), suggesting that hormonal cues may intersect with light signaling to fine-tune GST expression during floral development and pigmentation. The particularly dense accumulation of light‑responsive elements in Phi promoters resembles the “light‑sensing” promoter architecture described for anthocyanin biosynthetic genes and anthocyanin‑related GSTs in strawberry, grape, tree peony and other ornamentals, and is consistent with strong light‑ and stage‑dependent induction under defined UV‑A, blue, red and far‑red regimes [8, 35, 52]. Notably, Phi members harbored significantly more light-responsive elements per promoter than several other subfamilies, providing a cis-regulatory basis for subfamily-level differences in light sensitivity and supporting their prioritization as pigmentation-associated candidates. These observations suggest that, while the basic logic of “promoter cis‑module → tissue/stage‑specific expression → specialized GST function” is conserved, the detailed configuration of cis‑elements and their integration with light signaling pathways have been shaped by the ecological and evolutionary context of *Rhododendron* [21, 22, 43]. Nevertheless, cis-element prediction remains inferential; future promoter–reporter assays and transcription factor binding/perturbation experiments will be required to validate which motifs are functionally necessary for the observed light- and stage-dependent expression of key candidates.

The relationships between GST expression, anthocyanin accumulation and flower color provide further insights but also highlight the current limitations of our understanding [10, 23]. The broadly parallel changes in transcript levels of Phi and Tau candidates and total petal anthocyanin content under different light qualities support the view—established in apple, strawberry, grape, bayberry, tree peony and other species—that pigment‑related GSTs act as key modulators of vacuolar anthocyanin sequestration and, consequently, color intensity [16, 19, 20]. In *Rhododendron*, light quality appears to fine‑tune this module: shorter wavelengths tend to trigger rapid but transient responses, whereas red and far‑red light support more sustained anthocyanin accumulation, implying that different photoreceptors and downstream transcriptional regulators converge on a shared set of GST targets [30, 31, 35]. From an ornamental perspective, sustained anthocyanin accumulation under red/far-red light may contribute to deeper and/or more stable petal coloration during the transition from bud to fully open flowers. Compared with shorter wavelengths that often elicit rapid but transient responses, red/far-red may support a longer-lasting pigmentation trajectory, which is particularly relevant for display quality in *Rhododendron*. Although our evidence is correlative, these results suggest that adjusting the red/far-red proportion in controlled LED lighting could be explored to modulate flower color intensity during key developmental windows. At the same time, the present work remains largely correlative. Only total anthocyanins were quantified, without resolving individual anthocyanin species or co‑accumulating flavonoids, and functional evidence is currently limited to subcellular localization, which already reveals a more complex picture than predicted for some candidates (e.g. dual plasma‑membrane/nuclear localization of RhGSTF2) [11, 53].Temperature is an important co-determinant of anthocyanin metabolism and light responsiveness. To reduce potential confounding, all light-quality treatments in this study were conducted at a constant temperature (28 °C), so that treatment effects primarily reflect spectral differences rather than thermal variation. Nevertheless, temperature–light interactions were not explicitly examined here; future work incorporating factorial designs across multiple temperature settings will be valuable to assess the robustness and generality of the observed spectral effects. Future studies should therefore combine detailed metabolite profiling under contrasting light regimes with gain‑ and loss‑of‑function approaches—such as stable transformation, CRISPR/Cas‑mediated knock‑out and transient overexpression of key GSTs in *Rhododendron* and model plants—and with protein–protein interaction assays involving tonoplast transporters, transcription factors and photoreceptors [13, 14]. Such work will be essential to move from a correlative “light quality–GST expression–anthocyanin level” relationship to a mechanistic model in which light signaling, transcriptional regulation, GST‑mediated vacuolar transport and flower color phenotypes are integrated into a coherent network that can be exploited for molecular breeding and light‑based cultivation strategies in *Rhododendron* and related woody ornamentals [21, 22, 42]. 

## Conclusions

In this study, 87 GST family members were systematically identified in the *R. simsii* genome and designated *RsGST1–RsGST87* according to their physical positions on the chromosomes. Comprehensive analyzes of their amino acid sequences, gene structures and conserved elements allowed these genes to be classified into seven subfamilies: Tau, Phi, Lambda, Zeta, DHAR, TCHQD and Theta. Phylogenetic and genomic synteny analyzes indicated that expansion of the *RsGST* family was mainly driven by segmental duplication, whereas tandem duplication contributed only locally on certain chromosomal regions. Comparative synteny further showed that 14 pairs of orthologues were retained between *R. simsii* and its close relative *R. ovatum*, while only scattered collinear relationships were detected with *Arabidopsis*, suggesting that the core functional modules of the GST family have been preferentially conserved within the genus *Rhododendron.*

Promoter analysis revealed that the 2 kb upstream regions of *RsGST* genes are generally enriched in light-responsive and hormone-responsive cis-elements, implying that light signaling and endogenous hormones may act together to regulate their expression. qRT–PCR analyzes further demonstrated pronounced tissue specificity and light-quality-dependent differences in *RsGST* expression: flower buds were more sensitive to UV-A and blue light (365 nm, 460 nm), whereas fully open flowers responded more strongly to red and far-red light (660 nm, 730 nm); members with predominant expression in roots are likely to be involved mainly in antioxidant defense or detoxification. Consistent with these roles, CELLO-based prediction suggested that most Tau and Phi *RsGSTs* are cytoplasmic, while some Lambda, Zeta and DHAR members are targeted to chloroplasts or mitochondria, and transient expression of an *RhGSTF2* orthologue fused to GFP revealed a dual localization at the plasma membrane and in nuclei.

GFP fluorescence (green) was recorded using Ex 488 nm / Em 500–550 nm, and the red channel was recorded using Ex 633 nm / Em 650–750 nm. The coding sequence of *RhGST*F2 was fused in-frame to GFP under the control of the CaMV 35 S promoter (bottom row), and the empty pCAMBIA1300–GFP vector was used as a control (top row). Green channel shows GFP fluorescence; red channel shows red autofluorescence; merged images are shown in the right column.

By integrating cis-element profiles, expression patterns, synteny information and subcellular-localization data, *RsGSTF2*, *RsGSTL7* and *RsGSTU1* were highlighted as key candidate genes potentially involved in anthocyanin transport and/or stabilization. These findings provide new insights into the molecular mechanisms underlying flower color formation in Rhododendron, and lay a theoretical foundation for subsequent functional validation of candidate genes, breeding improvement and optimization of light-quality-based cultivation strategies.

## Supplementary Information

Supplementary Material 1.

## Data Availability

All data generated or analysed during this study are included in this published article and its supplementary information files.Supplementary Data 1: Figure6 qRT-PCR source data; Supplementary Data 2: Figure7 qRT-PCR source data; Supplementary Data 3: Figure9 qRT-PCR source data; Supplementary Data 4: Figure10 HPLC-DAD source data; Supplementary Figure S1: Morphological definition of petal developmental stages (S1-S4);Table S1: Basic information and physicochemical properties of 87 *RsGST* proteins in *Rhododendron simsii* ;Table S2: Subfamily-level comparison of physicochemical properties of *RsGST* proteins; Table S3: Ka, Ks and Ka/Ks values of duplicated *RsGST* gene pairs in *R. simsii* and orthologous pairs between *R. simsii* and *Arabidopsis* thaliana; Table S4: Summary and classification of cis-acting regulatory elements identified in the 2-kb upstream promoters of *RsGST* genes; Table S5: Primer sequences used for qRT–PCR analysis of representative *RhGST* genes; Table S6: Predicted subcellular localization of *RsGST* proteins in *Rhododendron simsii* based on CELLO v2.5;Table S7: Conserved domain architecture of the identified *RsGST* proteins in *Rhododendron simsii* ;Table S8: unanchored RsGST scaffoldIDs; Table S9: RsGST duplication collinearity MCScanX; Table S10: Consensus sequences of motifs 1–10 identified by MEME.

## Abbreviations

GST: Glutathione S-transferase
GSTs: Glutathione S-transferase proteins
RsGST: Glutathione S-transferase gene from Rhododendron sims
RhGST: Glutathione S-transferase gene from Rhododendron hybridum
Chr: Chromosome
Ka: Non-synonymous substitution rate
Ks: Synonymous substitution rate
Ka/Ks: Ratio of non-synonymous to synonymous substitution rates
RBH: Reciprocal best hit
qRT–PCR: Quantitative real-time polymerase chain reaction
HPLC–DAD: High-performance liquid chromatography with diode-array detection
FSW: Full-spectrum white light
UV-A: Ultraviolet A
