# Supplementary figures and images for: Genome-wide identification and light-quality-dependent characterization of Glutathione S-transferase genes involved in anthocyanin accumulation in Rhododendron
Source: BMC Plant Biol. 2026 Apr 2;26:843. doi: 10.1186/s12870-026-08698-1 (PMC13169737; doi:10.1186/s12870-026-08698-1)

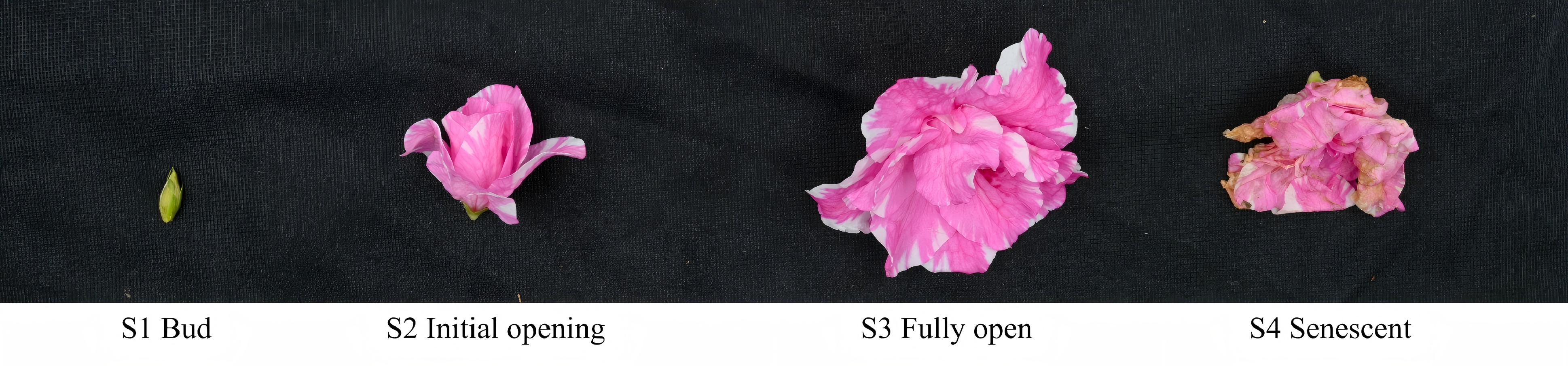

Supplement: Supplementary file 1 — Supplementary Material 1. [file 12870_2026_8698_MOESM1_ESM.zip › Supplementary File/Supplementary Fig S1 Morphological definition of petal developmental stages (S1–S4).jpg]
